# Harnessing next-generation microbial diagnostics to optimize infection management in immunocompromised hosts

**DOI:** 10.1097/QCO.0000000000001208

**Published:** 2026-06-17

**Authors:** Danielle Lee, Nicholas J. Norton, Adam P. Dale

**Affiliations:** a NIHR Southampton Clinical Research Facility and Biomedical Research Centre; b Department of Infection, University Hospital Southampton NHS Foundation Trust; c School of Clinical & Experimental Sciences, Faculty of Medicine, University of Southampton, Southampton, UK

**Keywords:** immunocompromised, infectious diseases, metagenomics, microbial diagnostics, next-generation sequencing

## Abstract

**Purpose of review:**

Conventional microbiological tests have limitations in the microbial diagnosis of immunocompromised patients. Next-generation sequencing (NGS) technologies have the potential to overcome some of these challenges by enabling rapid, comprehensive, and hypothesis-free pathogen detection, potentially improving the speed and accuracy of microbial diagnosis and subsequent clinical outcomes. This review summarizes current evidence for the use of NGS technologies in immunocompromised populations, highlights areas of demonstrated clinical impact, and identifies key priorities for broader clinical integration.

**Recent findings:**

Case reports and series have demonstrated the utility of NGS in diagnosing unusual or atypical infections amongst immunocompromised patients that were initially missed by conventional methods. Retrospective observational studies indicate that NGS can achieve higher sensitivity and greater pathogen detection rates than conventional diagnostics, although performance may be limited for certain pathogens, such as *Aspergillus* and *Mycobacterial* species. The clinical impact of NGS-guided interventions varies, reflecting both differences in study design and challenges in interpreting metagenomic data.

**Summary:**

NGS technologies have the potential to enhance microbial diagnosis in immunocompromised patients, particularly in complex, polymicrobial, or atypical infections where conventional methods fail. However, widespread clinical adoption is limited by high costs, complex workflows, and the need for advanced bioinformatics infrastructure and expertise. Further research is required to define clinical impact, cost-effectiveness, and to standardize workflows and guide optimal time for implementation, in order to inform evidence-based integration of NGS into routine clinical practice.

## INTRODUCTION

Immunocompromised patients are at increased risk of severe infection and infection-related mortality compared with their immunocompetent counterparts [1]. They are also at greater risk of antimicrobial resistant infections due to repeated prolonged courses of antimicrobials and frequent encounters with the healthcare system [1–3]. Timely and accurate microbiological diagnosis is therefore essential to guide prompt initiation of effective antimicrobial therapy, improve clinical outcomes, and to facilitate effective antimicrobial stewardship [4].

However, the diagnosis and management of infections in the immunocompromised can be challenging and may be delayed due to atypical clinical presentations and/or falsely reassuring biomarkers of inflammation/infection [5,6]. The diagnostic challenge is further complicated by the broad array of microorganisms that can cause infection in the immunocompromised, including infections secondary to low pathogenicity or ‘atypical’ bacterial, viral, fungal or protozoal species, and given the increased risk of polymicrobial infections [1,7,8].

The use of conventional microbiological diagnostic tests, including those underpinned by culture-, serology- and polymerase chain reaction (PCR)-based technologies, has important limitations in the immunocompromised. First, they are targeted to specific infectious agents and restricted in scope; for example many atypical or fastidious organisms are difficult to culture [9], hence positive detection requires the requesting clinical team to have included the causative pathogen in their differential diagnosis so that the appropriate test is sent. Second, in the case of culture, prior initiation of active antimicrobial therapy significantly decreases diagnostic yield [10,11]. Third, the performance of serological tests can be significantly impaired in patients treated with cell depleting therapies and lead to false negative results secondary to impaired antibody response generation following infection [12,13]. Fourth, many conventional tests are limited by prolonged turnaround times, particularly for culture-based methods or where specialist testing is centralized at reference laboratories [14,15]. This issue is further compounded by delays in requesting tests for rarer microbiological causes of specific infection syndromes, with second- or third-line testing only initiated when poor response to initial empirical therapy is documented, or where other investigations, for example cross-sectional imaging, suggest an atypical infectious cause. The resultant diagnostic delay frequently leads to prolonged and repeated use of empiric broad-spectrum antimicrobials, while increasing the risk of undertreating patients with rarer infection syndromes and contributing to subsequent poor outcomes [16–18].

Next-generation sequencing (NGS) technologies, also known as high-throughput or massively parallel sequencing, have the potential to overcome some of the limitations associated with the use of conventional microbiological diagnostics in the immunocompromised. Utilisation of these technologies in this setting has developed rapidly over the past 10–20 years and enables the direct, simultaneous sequencing of nucleic acid within a sample, providing a culture-independent method with potential to detect all microorganisms present [19]. This allows for untargeted hypothesis-free testing of samples for multiple pathogens simultaneously without requiring prior knowledge of the suspected infectious agent [20]. This is particularly valuable for increasing the precision and sensitivity of diagnosing rare, atypical and polymicrobial infections to which immunocompromised individuals are especially susceptible [21–24]. In addition to pathogen detection and classification, NGS offers the ability to extract additional data from the sequences obtained, including detection of antimicrobial resistance genes [25]. For clinicians, the rapid detection of drug resistance genes, including from mobile genetic elements, is a powerful tool to enable early optimization of antimicrobial therapy. The reconstruction of pathogen genomes allows phylogenies to be inferred which can identify transmission events and outbreak clusters which may go unrecognized by conventional techniques [26].

However, there are an array of challenges and barriers to the more widespread implementation of these tools in routine clinical diagnostic practice, including but not limited to: technical barriers associated with sample optimization, including removal of background environmental and host contaminant DNA signal [27,28]; standardization of assays and associated laboratory-based quality assurance and accreditation procedures [29]; generation of high-quality trial data to demonstrate the impact and added value of integrating these technologies within microbial diagnostic pipelines [30]; and limited understanding amongst clinical teams regarding data analysis and interpretation [31,32].

In this narrative review, we outline the various NGS technologies utilized in clinical microbial diagnostics and evaluate their respective advantages and limitations. We then summarize the clinical evidence supporting the use of NGS in immunocompromised patients, highlighting examples of demonstrated clinical impact. Finally, we discuss the key barriers to implementation and identify priority areas for future development to facilitate the broader integration of these technologies into the care of immunocompromised patients. 

**Box 1:**
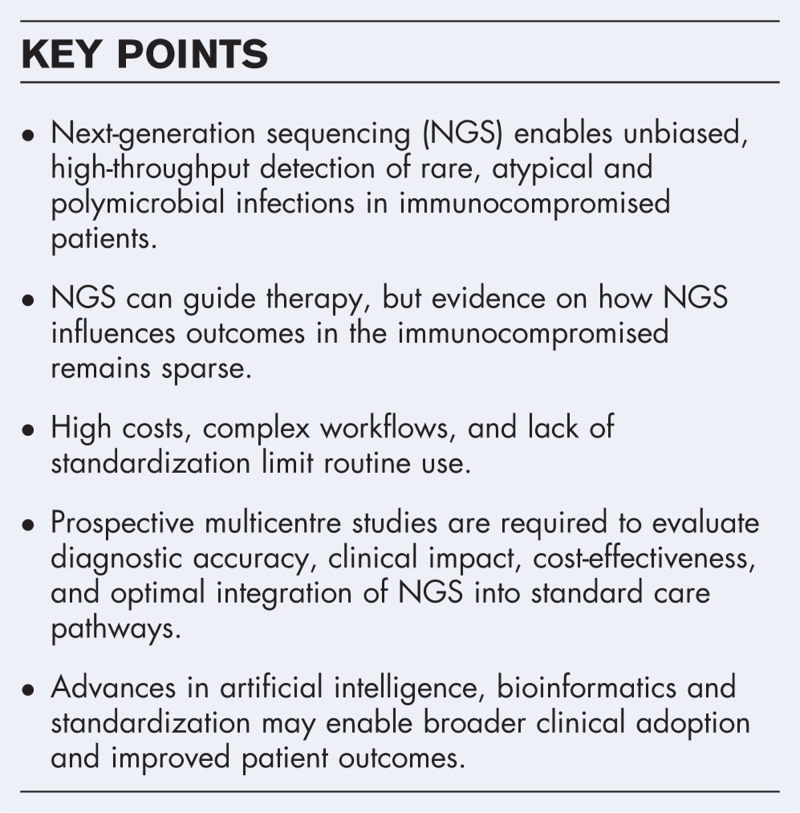
no caption available

## OVERVIEW OF NEXT-GENERATION SEQUENCING TECHNOLOGIES

**FIGURE 1:**
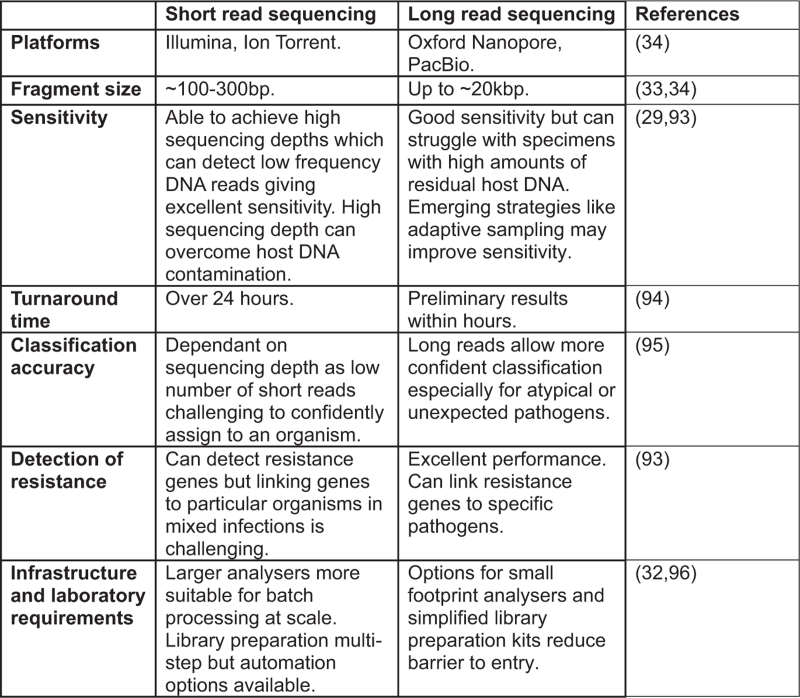
Comparison of next-generation sequencing approaches.

NGS involves the parallel sequencing of large numbers of DNA or RNA molecules. Two broad approaches have emerged; short-read sequencing which produces millions of sequences typically 100–300 bp in length, and long-read sequencing which produces fewer sequences but of lengths up to ~20 kb [33,34]. A comparison of each of these approaches is summarized in Fig. 1. NGS can be utilized in the laboratory on cultured isolates to produce whole genome sequences or to detect resistance genes. When applied to patient samples, NGS is usually deployed as metagenomic sequencing, that is analysis of all genetic material present regardless of source organism. In brief, metagenomic NGS (mNGS) involves extracting nucleic acid fragments from a sample, sequencing them using high-throughput sequencing platforms, and then comparing these to databases of known microbial sequences to characterize the presence of microorganisms within the sample [35].

Clinical samples typically contain large amounts of human genetic material. The abundance of host reads makes detection of rare microbial reads challenging and can overwhelm the microbial signal, subsequently reducing sensitivity of mNGS in detecting these microorganisms [36,37]. Most clinical approaches involve a host-depletion step where human cells are lysed and the resulting nucleic acid removed enzymatically [38]. However, this process also carries the risk of negatively impacting on microbial detection through inadvertent depletion of microbial DNA, especially for intracellular or structurally fragile microorganisms [28,39], potentially leading to false-negative results.

Targeted next-generation sequencing (tNGS) approaches can also be applied to reduce interference from background host and environmental nucleic acids. This involves the amplification and enrichment of a ubiquitous conserved gene across all microorganisms in a particular microbial kingdom, such as the 16S rRNA gene in bacteria, from a sample before sequencing [21]. Enrichment of these target genes is done using either multiplex PCR amplicon-based or hybrid capture-based methodologies, which increases the concentration of low-abundance or fastidious microorganisms, thus enhancing detection sensitivity [40,41]. Whilst this is associated with reduced background interference, sequencing costs and data burden, the scope of microbial detection is limited compared with unbiased mNGS approaches, as prior knowledge of the suspected pathogen group is required [40,42].

## NEXT-GENERATION SEQUENCING IN IMMUNOCOMPROMISED PATIENTS: CLINICAL INSIGHTS

A multitude of clinical studies assessing the impact of NGS technologies for microbial detection in the context of immunocompromised patients have been published over the last 5 years, the vast majority being retrospective in nature and focused on lower respiratory tract infections. However, a small number of studies have been conducted outside of respiratory infection, including those applying NGS technologies to assess more broadly for the microbial aetiology underpinning central nervous system and bloodstream infections.

The diverse range of microorganisms with the propensity to cause lower respiratory tract infections in immunocompromised patients poses a major diagnostic challenge, with delayed or missed diagnosis potentially leading to poor clinical outcomes if empirical antimicrobial therapy is inadequate [7]. Current microbiological diagnostic strategies applied in these patients are complex and rely on a combination of culture, serological and PCR-based methodologies, while advanced testing for rare pathogens requires reference laboratories, resulting in prolonged turnaround times [43].

Clinical studies performed in the context of both heterogenous and focused subsets of immunocompromised patients have assessed the effectiveness of NGS-based technologies, including mNGS and tNGS, in their ability to detect the likely microbial aetiology of lower respiratory tract infection [44,45,46^▪^,^47^–^56^]. These studies demonstrate that NGS performed on bronchoalveolar lavage (BAL) effectively detects both mono- and polymicrobial pneumonic infections in immunocompromised patients, with substantially higher sensitivity than culture, although interpreting the clinical significance of low-pathogenicity microbial reads remains challenging [57,58].

A significant methodological limitation of the cited studies is that many compare NGS technologies against sub-optimal standard diagnostic methods, as opposed to comparing performance against more sensitive diagnostic tests used commonly in the high income setting to confirm or exclude high-priority pathogens [7,43,59,60]. Where studies have compared the diagnostic performance of NGS technologies against gold standard high sensitivity diagnostic tests, the results have been variable. For example, in the context of persons living with HIV, Miao *et al.* assessed the performance of mNGS for the diagnosis of pulmonary mycobacterial infection as compared with mycobacterial culture and Xpert MTB/RIF PCR in a retrospective study inclusive of 146 eligible participants. Sensitivity of mNGS to detect microbiologically proven pulmonary mycobacterial infection, *M. tuberculosis* infection or nontuberculous mycobacterial infection was 78.8% [95% confidence intervals (CI) 62.2–89.3], 76.9% (49.7–91.8) and 72.2% (49.1–87.5), respectively [61]. In a second retrospective study, Xu *et al.* assessed the performance of mNGS in diagnosing invasive pulmonary aspergillosis (IPA) amongst 589 haematological patients with or without a diagnosis of invasive pulmonary aspergillosis, as determined by reference to international diagnostic guidelines [62]. Similar to the findings outlined above for mycobacterial infection, pooled analyses demonstrated that mNGS was less sensitive than PCR for the diagnosis of IPA using BAL (59.6% [44.3–73.3] vs. 83.0% [68.7–91.9]). Interestingly, however, mNGS performed similarly to PCR when utilized on blood (55.0% [46.7–63.0] vs. 51.7% [43.4–59.8]) [63^▪^]. Finally, and in contrast to the previously mentioned studies, Luo *et al.* recently assessed the diagnostic performance of nanopore-based mNGS in a prospective cohort of 118 lung cancer patients presenting with clinically suspected *P. jirovecii* infection and demonstrated that the mNGS approach utilized detected a higher number of *P. jirovecii* infections as compared with methenamine silver staining and PCR (33.0%, 4.2% and 30.5%, respectively), with 100% sensitivity and specificity [64]. These data demonstrate that, while NGS approaches show significant promise in detecting a broad array of respiratory pathogens in the immunocompromised, there are limitations in sensitivity of detection for some critical respiratory pathogens as compared with targeted PCR.

A large number of studies have evaluated the use of mNGS for diagnosing central nervous system infections across heterogenous patient groups and have demonstrated this is a valuable adjunct for identifying the causative pathogen [65,66]. Although many of these studies include immunocompromised patients within their cohorts, dedicated evaluations for the diagnostic performance of NGS-based approaches specifically in immunocompromised populations remain limited in both number and sample size [67–70]. Utilization of NGS-based approaches has been evaluated on blood samples in immunocompromised patient cohorts, including amongst those with febrile neutropenia, and although pathogen detection is generally increased in this setting, the clinical impact of the results generated from such tests remains understudied [71–73].

Although mNGS-based tests allow for increased microbial detection across a range of sample types [23,74,75], evidence assessing whether this translates into achieving positive clinical outcomes for patients is less consistent. Case studies in particular have highlighted the strengths of using mNGS to diagnose polymicrobial infections [76–78] and rare, unusual infections in the immunocompromised [79,80,81,82^▪^], where conventional microbiological techniques failed to make a diagnosis or resulted in an initial misdiagnosis, leading to subsequent initiation of appropriate targeted antimicrobial treatment with clinical improvement. For example, Huang *et al.* described a case where mNGS was used to identify *Coxiella burnetii* causing chronic Q fever in a patient receiving dialysis for chronic kidney disease who had initially been misdiagnosed with metastatic malignancy and tuberculosis via conventional tests, and who subsequently demonstrated sustained clinical and radiological improvement after commencing targeted doxycycline therapy [82^▪^]. For the diagnosis of polymicrobial infections, Zhao and Ye described a case where mNGS of BAL fluid was successfully used to diagnose a polymicrobial cause of severe community acquired pneumonia in an immunocompromised patient on long-term rituximab and corticosteroids for IgA nephropathy, who subsequently made a full recovery after commencing appropriate targeted antimicrobial therapy [77].

Although a number of studies have shown that the use of mNGS resulted in positive clinical impacts in both immunocompetent and immunocompromised patients, such as guiding the initiation or escalation of appropriate targeted therapy, or the discontinuation of unnecessary broad-spectrum antimicrobials [22,23,73], other observational studies have shown only limited clinical impact. For example, a systematic review and meta-analysis which assessed the diagnostic accuracy of the commercially available Karius plasma cell-free mNGS (cf-mNGS) test in a real-world clinical setting, for which the majority of patients from the included studies were immunocompromised, concluded that cf-mNGS only had minimal influence on the likelihood of the presence of infection, which could reflect the observation that, in clinical practice, mNGS is usually only utilized when conventional tests fail to provide a diagnosis, therefore resulting in a lower pretest probability of infection [31]. Recent retrospective observational studies assessing the clinical performance of this test in immunocompromised patients also demonstrated limited clinical benefits, resulting in a change in clinical management in only a minority of cases, some of which resulted in negative outcomes of unnecessary diagnostics and/or treatment [72,83].

## LIMITATIONS AND BARRIERS TO IMPLEMENTATION OF NEXT-GENERATION SEQUENCING TECHNOLOGIES IN ROUTINE CLINICAL PRACTICE

Despite some of the advantages of NGS technologies described here, there continue to be barriers and limitations to the more widespread implementation of these technologies in routine clinical practice. As discussed previously, the major barrier relates to the need to generate high-quality data from well controlled diagnostic trials designed to define and quantify the clinical impact and cost effectiveness of NGS-based testing across diverse clinical settings. The relatively high cost of NGS testing is often cited as a key barrier to its broader adoption in routine clinical practice, particularly in low- and middle-income countries [84]. In real-world settings, mNGS can cost up to $3000 per sample, substantially exceeding the cost of conventional diagnostic testing methods such as in-house PCR ($5–10 per sample) and blood cultures (<$50 per sample) [84,85]. Although these costs are expected to decline with technological advances [20], the current cost-effectiveness of using these technologies in routine clinical practice remains uncertain. This is partly because immunocompromised patients are typically treated with broad-spectrum antimicrobials at the onset of infection, which often already target the pathogens detected by mNGS [22]. As a result, mNGS may be most appropriately reserved for complex cases where conventional diagnostics have failed to establish a diagnosis [84]. Further studies are needed to evaluate cost-effectiveness, particularly in balancing upfront costs of NGS against potential savings from reduced hospital stays and fewer complications associated with delayed diagnosis and treatment [30,86].

Another key limitation of NGS is its higher false-positive rate for pathogen detection, which stems from its ability to identify all microorganisms in a sample in an unbiased manner, including those which may only be commensals or contaminants. NGS cannot distinguish between microorganisms which are pathogenic, commensals or contaminants, or remnants from previous infections [87]. This challenge is particularly pronounced in immunocompromised patients, such as those with malignancies undergoing chemotherapy, who are more susceptible to mucosal barrier injury due to both immune dysfunction and the cytotoxic effects of treatment [88]. As a result, microbial DNA from normal gut or oral flora may translocate across compromised mucosal surfaces and be detected by NGS, increasing the likelihood of false-positive findings and subsequent inappropriate investigatory or treatment interventions [89].

The complexity of NGS workflows presents an additional barrier to their integration into routine clinical practice. These processes require advanced bioinformatics expertise and statistical proficiency to manage and analyse the large volumes of data generated, as well as specialized laboratory infrastructure to support the computational and data storage demands involved [32,90]. The lack of standardization and clear guidelines for NGS workflows and data analysis further complicates interpretation, making results more challenging for clinicians to apply in practice [32]. Moreover, NGS workflows are susceptible to contamination and bias at multiple stages, necessitating rigorous sample preparation and quality control measures to ensure accuracy [35,91]. Emerging advances in artificial intelligence, including machine and deep learning, offer promising solutions to these challenges. These technologies have the potential to streamline metagenomic sequencing workflows by improving quality control, enhancing sequence assembly and reconstruction, identifying and correcting sequencing errors, and enhancing refinement and evaluation tools used to optimize completeness while minimizing contamination of sequenced genomes [92].

## CONCLUSION AND FUTURE DIRECTIONS

NGS technologies offer substantial potential to improve the diagnosis of infections in immunocompromised patients, particularly in cases where conventional microbiological approaches are limited. Evidence from case reports, case series, and observational studies highlight the strengths of NGS in detecting rare, atypical and polymicrobial infections, enabling timely initiation of targeted antimicrobial therapy and, in some instances, improving clinical outcomes. These capabilities are particularly valuable for immunocompromised patients, who are more susceptible to infection by low-pathogenicity microorganisms and may present with atypical clinical features.

Despite these advantages, the current evidence base is largely retrospective, with few prospective, multicentre studies directly assessing the clinical impact of NGS in the immunocompromised. Limitations in sensitivity for some key pathogens, high rates of false positives, and challenges in interpreting complex metagenomic data highlight the need for further rigorous evaluation. High costs, technical complexity, and limited accessibility also constrain broader clinical adoption. The absence of universally accepted reference standards in some infectious diseases contexts further complicates the design of diagnostic accuracy studies, introducing potential biases in outcome assessment.

Future studies should prioritize generating high-quality, prospective evidence that evaluates both the diagnostic and clinical utility of NGS across diverse patient populations and infection types. Studies assessing cost-effectiveness and the impact on patient outcomes are critical. Advances in standardization, bioinformatics, and artificial intelligence offers the potential to address current technical and interpretive barriers, improving workflow efficiency, reducing contamination, and enhancing accuracy. By addressing these challenges, NGS could become an increasingly integrated component of precision microbial diagnostics that could ultimately enable more rapid, accurate and personalized care for immunocompromised patients with infection.

## Acknowledgements

*None.*

### Financial support and sponsorship

*None.*

### Conflicts of interest

*A.P.D. has received grant funding from ILiAD Biotechnologies and Moderna Ltd and has performed consultancy work for GSK. He receives no personal financial payment for this work*

## REFERENCES AND RECOMMENDED READING

Papers of particular interest, published within the annual period of review, have been highlighted as:

▪ of special interest
▪▪ of outstanding interest
